# Conduction Aphasia as Initial Manifestation of Tuberculous Meningitis

**DOI:** 10.7759/cureus.2889

**Published:** 2018-06-27

**Authors:** Miguel A Garcia-Grimshaw, Francisco A Gutierrez-Manjarrez, Alejandra Gonzalez-Duarte

**Affiliations:** 1 Internal Medicine, Hospital General De Tijuana, Tijuana, MEX; 2 Neurology, Hospital General De Tijuana, Tijuana, MEX; 3 Neurology, Instituto Nacional De, Mexico City, MEX

**Keywords:** conduction aphasia, tuberculous meningitis, stroke, aphasia, mycobacterium tuberculosis

## Abstract

Conduction aphasia being the arcuate fasciculus of the site of structural injury is a speech disorder characterized by fluent, spontaneous speech and paraphasias, intact auditory comprehension, and limited repetition. One of the causes of stroke in young adults is the Mycobacterium tuberculosis (MTB) infection, which may cause cerebral ischemia secondary to artery obliteration. In this case report, we present a previously healthy 24-year-old woman that presented with a sudden onset of aphasia; MTB was identified as the etiological agent. Tuberculous meningitis (TBM) has a wide range of clinical manifestations with aphasia being one of the rarest forms of initial presentation.

## Introduction

Aphasia is a speech disorder secondary to a brain lesion mainly localized on the cerebral cortex. Conduction aphasia (CA) is characterized by fluent spontaneous speech, paraphasias, intact auditory comprehension, and limited repetition. The lesion is located in the arcuate fasciculus of the dominant hemisphere [1]. The first documented case of aphasia due to tuberculous meningitis (TBM) was described by Schutz in 1881 [2]. Currently, Mycobacterium tuberculosis (MTB) remains a frequent cause of neuroinfection, with a broad range of clinical signs [3].

## Case presentation

**Figure 1:**
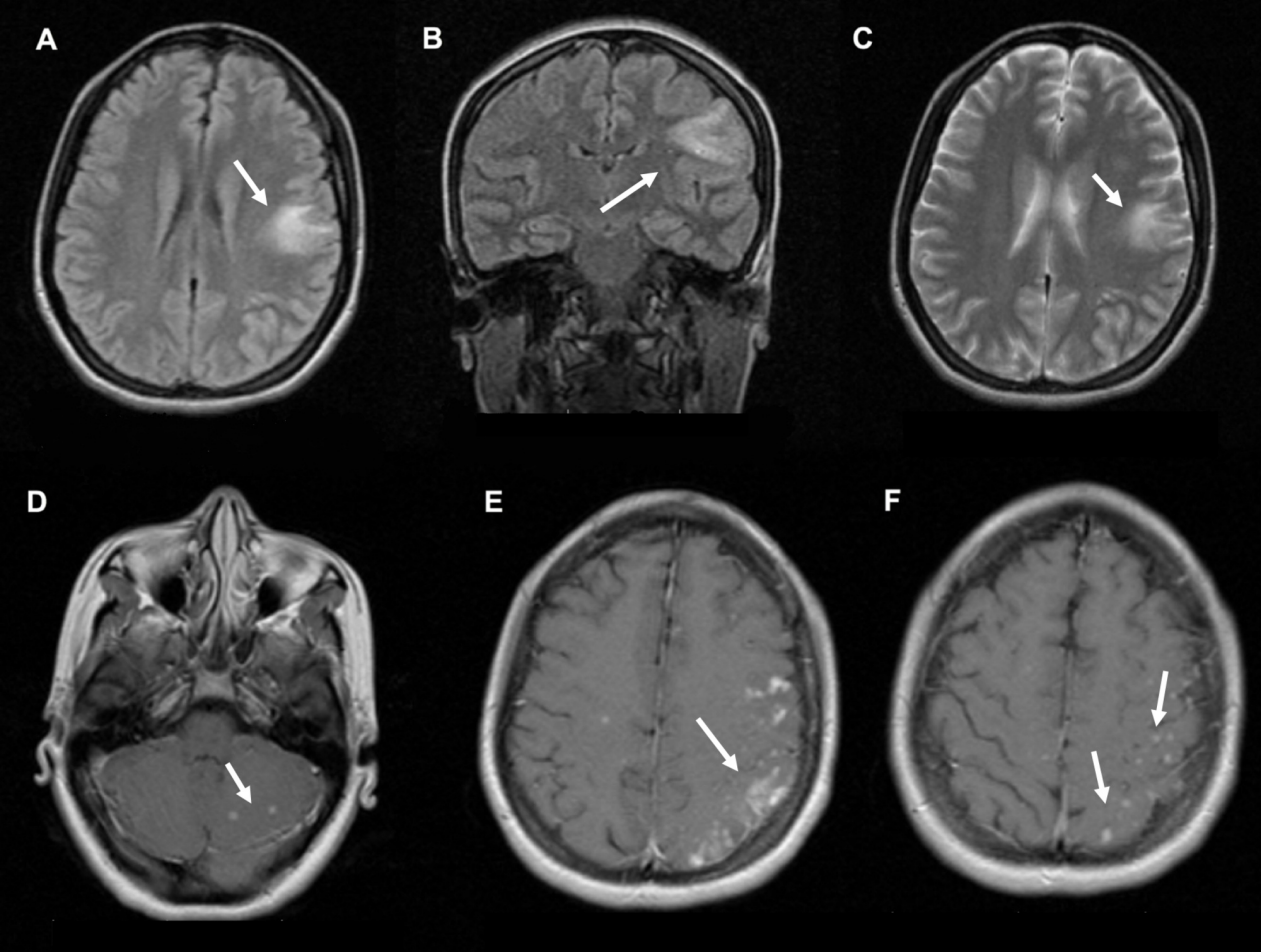
Case magnetic resonance image (MRI) (A) Axial MRI in FLAIR sequence showing a left temporal hyperintense lesion of the white matter in the left temporal lobe. (B) Coronal FLAIR sequence with inferior temporal hyperintense lesion of the white matter. (C) Axial T2-weighted sequence with left temporal hyperintensity. (D) Axial contrast-enhanced T1-weighted sequence showing nodular enhancing lesions on the cerebellum. (E) Axial contrast-enhanced T1-weighted sequence with cortical and nodular enhancing lesions in the left temporal lobe. (F) Axial contrast-enhanced T1-weighted sequence showing cortical nodular enhancing lesions. FLAIR: fluid-attenuated inversion recovery.

A previously healthy right-handed 24-year-old woman developed a headache three days before admission in the left frontal region with an 8/10 intensity accompanied by retro-ocular pain and phosphenes. Twenty-four hours later, she developed a speech disorder and was presented to the emergency department. Upon arrival, her blood pressure was normal (110/70 mmHg), tachycardic with a heart rate of 94 beats per minute, normal respiratory rate (14 breaths per minute) and temperature (36.2°C). The neurological examination showed normal mental status, with fluent speech and no paraphasias. The patient had normal nomination, and she was able to understand and obey simple commands. She was able to read-out loud and write, but could not repeat simple phrases; the rest of the examination was normal. Her blood work revealed hemoglobin of 8.9 g/dL and 4,390 leukocytes mm^3^/mL, human immunodeficiency virus type one and two antibodies detection were negative; rest of the blood work was normal. A chest X-ray was performed and it revealed generalized symmetrical interstitial infiltrates. Acontrast-enhanced magnetic resonance image (MRI) of the brain showed multiple edematous nodular lesions in the left parietal lobe and cerebellum on the T1-weighted sequence (Figure 1).

Transthoracic echocardiogram, carotid and vertebral Doppler ultrasound examination were normal. Cerebrospinal fluid (CSF) analysis showed 88 cells mm3/mL of which 65% were mononuclear with low glucose of 36 mg/dL, a central glucose of 116 mg/dL (ratio 0.31), and elevated proteins of 201 mg/dL. The CSF smear was negative and Gene Xpert (Cepheid Inc., Sunnyvale, CA, USA) MTB/rifampicin (RIF) in the CSF was positive for MTB. She was started on first-line antituberculosis drugs (isoniazid, rifampicin, pyrazinamide and ethambutol) and dexamethasone (0.4 mg/kg/day). The patient was discharged on the 11th day of hospital stay. After her three-month follow-up, she still had CA.

## Discussion

Based on the clinical examination of this case, the speech disorder was classified as a CA, the latter is characterized by alterations on the repetition of simple phrases, having a normal fluency and sometimes with paraphasias and episodes of self-correction; comprehension is normal, and the patient is able to understand a conversation; nomination may be limited. Writing and reading abnormalities are not always present. The lesioned site of the brain is the arcuate fasciculus, which is a neuronal tract that travels through the depth of the temporal lobe surrounding the Sylvian fissure [1,4]. In this patient, we identified MTB as the etiologic agent that caused the structural lesion.

TBM is the most frequent form of active tuberculosis in the central nervous system (CNS). A typical case presents with low-grade fever, weight loss, and headache with an onset time of 10 days on average and clinical signs such as neck stiffness and raised intracranial pressure, altered level of consciousness with a Glasgow Coma Scale (GCS) score of 10 points on average; as the disease progresses the patient may show focal neurological signs, cranial nerve paralysis, seizures and cerebral infarcts in up to 30% of all cases.

In the lumbar puncture, raised intracranial pressure is detected in up to 50% of the patients. CSF analysis showed leukocytosis of mononuclear predominance in a range of 100-1000 cells mm^3^/mL, elevated protein count in ranges of 100-500 mg/dL, low glucose with a CSF/serum glucose ratio less than 0.5 in 95% of cases; CSF smear being positive only in 10%-20% of the cases. Molecular methods like the Gene Xpert MTB/RIF, have been approved as the first diagnostic test to perform in the CSF upon clinical suspicion, having a variable sensitivity ranging between 67%-73% and specificity of 94%-99.5%.

The most common neuroimaging findings are the following: hydrocephalus 45%, cerebral edema and ischemia between 8%-44%, basal meningeal reinforcement 8%-34%, tuberculoma 8%-31%, and cerebral nodules with enhancement. The prognosis depends on their neurological status at the moment of diagnosis, stage of the disease, diagnostic delays, and hydrocephalus; mortality ranges from 7%-65% in developed countries and up to 69% in low resources countries and up to 50% of surviving patients remain with significant neurologic sequelae [5-6].

## Conclusions

Mycobacterium tuberculosis infection of the CNS is a frequent and highly underdiagnosed disease which can present with a broad range of typical and atypical signs. CA is one of the rarest forms of initial manifestations that clinicians should be aware of in order to make quick therapeutic decisions.
